# Different solvents and organic modifiers for the control of crystallographic parameters in nano-crystallite hydroxyapatite for amplification of photocatalytic activity

**DOI:** 10.1039/d3na01122d

**Published:** 2024-04-10

**Authors:** Md. Kawsar, Md. Sahadat Hossain, Sumaya Tabassum, Newaz Mohammed Bahadur, Samina Ahmed

**Affiliations:** a Glass Research Division, Institute of Glass & Ceramic Research and Testing, Bangladesh Council of Scientific and Industrial Research (BCSIR) Dhaka-1205 Bangladesh shanta_samina@yahoo.com; b Department of Applied Chemistry and Chemical Engineering, Noakhali Science and Technology University Noakhali Bangladesh; c BCSIR Dhaka Laboratories, Bangladesh Council of Scientific and Industrial Research (BCSIR) Dhaka-1205 Bangladesh

## Abstract

In this research, HAp nanocrystals were synthesized using conventional wet chemical precipitation methods using various organic modifiers, including urea, palmitic acid, and naphthalene. Ethanol and isopropyl alcohol (IPA) were used as solvents in this process. Different characterization techniques, namely X-ray diffraction (XRD), scanning electron microscopy (SEM), and UV-vis absorption spectroscopy, were employed to ascertain the formation of HAp nanocrystals. Numerous structural parameters, including lattice parameters, unit cell size, volume of the unit cell, specific surface area, degree of crystallinity, dislocation density, macrostrain, and crystallinity index, were assessed using XRD data. The linear straight-line method of Scherrer's equation, Monshi–Scherrer's method, the Williamson–Hall method, the size–strain plot method, the Halder–Wagner method, and Sahadat–Scherrer's model were applied to compute the crystallite size of the synthesized HAp samples. All the synthesized HAp has crystalline structures within the permissible range of 1–150 nm which were estimated from the XRD data using the mentioned models. However, the values for strain (from −3 × 10^−4^ to 6.4 × 10^−3^), strain (from −9.599 × 10^4^ to 7 × 10^10^ N m^−2^), and energy density (from −11 × 10^11^ to 2 × 10^7^ J m^−3^) were also calculated for the synthesized samples. In addition, the optical band gap energy of the synthesized HAp was computed (5.89 to 6.19 eV). The synthesis media have a control on the crystallographic planes, *e.g.* in the case of the ethanol medium, the (110) plane exhibited significant intensity (which could potentially serve as a driving force for enhancing photocatalytic activity). The use of 100% ethanol HAp yields the most favorable outcome regarding both the degradation percentage (91.79%) and degradation capacity (7%) for the Congo red dye.

## Abstract 2

Certain crystal plane was develop in hydroxyapatite using organic modifiers/solvents to enhance photocatalytic activity.

## Graphical abstract

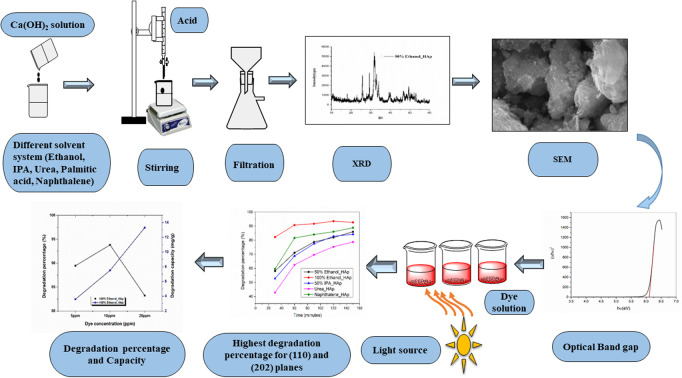

## Introduction

Calcium phosphate, scientifically referred to as Ca_10_(PO_4_)_6_(OH)_2_ and commonly recognized as hydroxyapatite (HAp), holds significant promise as a vital component for artificial bone tissue engineering, primarily because of its remarkable biocompatibility, osteoconductivity, hemocompatibility, and photocatalytic activity.^1,2^ Numerous methods, namely the wet chemical precipitation method,^3,4^ mechanochemical method,^5,6^ hydrothermal method,^7,8^ solid-state method,^3,9^ and sol–gel method^10,11^ coupled with several other methods have already been used to synthesize HAp nanoparticles. However, crystallographic properties, which include lattice parameters, volume of the unit cell, crystallite size, degree of crystallinity, dislocation density, crystallinity index, *etc.* are essential features of HAp.^12–14^ In particular, the size of particles, principally unit cell dimensions, significantly impacts materials' physical and chemical properties. Hexagonal HAp comprises *P*6_3_/*m* space groups by a sixfold symmetric axis arranged with a three-dimensional helix as well as a reflecting plane, whereas the usual lattice parameters are: (1) *a* = *b* = 9.42 Å and *c* = 6.88 Å; (2) cell volume = 530.301 (Å);^3^ and (3) crystal density = 3.140 g cm^−3^.^15,16^ Nanocrystals show intrinsic strain arising from size internment that may be modified by adjusting synthesis factors such as pH and concentration, which impact optical as well as other features.^17^ The photocatalytic properties of HAp have been under scrutiny due to its use with metals for dye degradation. Despite the use of both HAp and metal oxide, HAp alone has been investigated for photocatalysis for dye degradation.^3^ Stable substances, such as colors from the textile or dye industries, pose a significant threat to human health when entering aquatic systems. Therefore, removing color from wastewater is necessary.^18,19^ Semiconducting-based photocatalysis is an effective technology for the critical degradation and mineralization of organic pollutants. It offers outstanding advantages such as adequate organic matter mineralization into water, carbon dioxide, and other mineral ions, no secondary pollutant production, and uses sunlight as a cheap irradiation source when semiconductor band gap energy is in the visible region.^20–22^ Typically, semiconducting-based photocatalysis utilizes four reactive species, such as photogenerated electrons and holes, superoxides, and hydroxyl radicals, to degrade pollutants in an aqueous medium.^23,24^ Here, the focus lies in the estimation of different nanocrystallite sizes of synthesized HAp from their X-ray diffraction (XRD) data by employing the linear straight-line method of Scherrer's equation, Monshi–Scherrer's method, the Williamson–Hall method, the size–strain plot method, the Halder–Wagner method, and Sahadat–Scherrer's model. Additionally, the Williamson–Hall method consists of a uniform deformation model (UDM), uniform stress deformation model (USDM), and uniform deformation energy density model (UDEDM), which includes stress, strain, and energy density correspondingly, for estimating several elastic variables. Furthermore, in the size–strain plot method, the widened portion denotes the Lorentzian function and the broadened part is considered the Gaussian function in XRD peak profile analysis. This method is preferable to the Williamson–Hall model due to its high accuracy and high precision of XRD peaks, which are high.^25–27^ Conversely, the physical peak widening is considered the Voigt function and average size and strain can be measured from XRD peak broadening in the Halder–Wagner method.^28^ Moreover, Sahadat–Scherrer's model is like Scherrer's model, but there is a little difference, where the intercept goes through the origin. The wet chemical precipitation method is a cost-effective and simple method for synthesizing hydroxyapatite nanoparticles. It offers high purity, low reaction time, and scalability, making it suitable for large quantities. It also allows control over sintering time, temperature, acidity, and solvent volume, resulting in nano- and micrometer structures. Unlike other methods, it offers better control, reproducibility, and cost-effectiveness.^29,30^

In this study, HAp was synthesized *via* the wet-chemical precipitation method, where different solvent systems such as ethanol, isopropyl alcohol, and distilled water are used. Apart from that, several organic modifiers such as urea, palmitic acid, and naphthalene were utilized to modify the structure of HAp nanocrystals. Then the synthesized HAp was subjected to study the photodegradation of Congo red dye in an aqueous solution.

## Materials and methods

### Materials

To conduct this experiment, phosphoric acid (H_3_PO_4_), calcium hydroxide (Ca(OH)_2_), absolute ethanol (C_2_H_5_OH), isopropyl alcohol (IPA) (C_3_H_8_O), urea (CH_4_N_2_O), palmitic acid (C_16_H_32_O_2_), naphthalene (C_10_H_8_) and distilled water (DI) were used. All of these chemicals are of analytical grade, and these chemicals, except DI, were purchased from E-Merck Germany. No buffer solution was utilized in this experiment, but ammonium hydroxide (NH_4_OH) and nitric acid (HNO_3_) were used to maintain the pH (10–11) of the reaction media.

### Methods

#### Synthesizing HAp with different solvent systems

For synthesizing HAp nanoparticles, a calcium and phosphate molar ratio of 1.67 was maintained. First, a predetermined amount of Ca(OH)_2_ of 1.67 M was prepared using 30 mL DI. The solution was then introduced in two distinctive solvent systems: ethanol (50 and 100% (v/v)) and IPA 50% (v/v). Finally, the solution was stirred at 350 rpm for 2 h. The calculated amount of H_3_PO_4_ (1.0 M) was added dropwise from the burette, maintaining the rate at 3 mL min^−1^. While adding H_3_PO_4_ into the reaction vessel, the pH of the reaction was maintained at 10–11 by incorporating NH_4_OH solution. To complete the reaction, continuous stirring was applied. After completing the reaction, the precipitate was separated using Whatman-44 filter paper. Subsequently, the product was dried in an oven at 105 °C for 24 h.^31^ The full procedure was described elsewhere.^32^

#### Synthesizing HAp with different organic modifiers

**Fig. 1:**
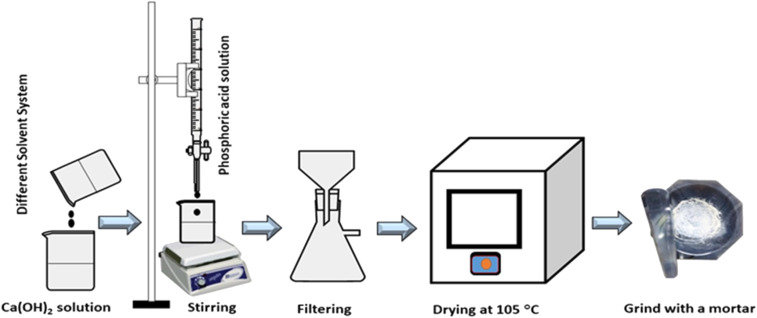
Flow diagram of synthesizing HAp using different solvent systems.

To synthesize HAp with different organic modifiers, a stoichiometric amount of (Ca/P = 1.67) 1.67 M Ca(OH)_2_ was prepared using 50 mL DI water and the solution was stirred at 300 rpm for 2 h. Subsequently, different amounts of organic modifiers (urea (0.33 M), palmitic acid (0.077 M), and naphthalene (0.15 M)) were added to the reaction vessel (500 mL beaker). A predetermined amount of 1.0 M H_3_PO_4_ was added dropwise from the burette, and the pH of the solution was maintained at 10–11 using NH_4_OH. After retaining the pH, the solution was subjected to further stirring for 2 h to accomplish the reaction. Finally, the precipitate was separated using Whatman filter paper-44 and dried in an oven for 6 h at 105 °C.^33^ The process is presented here in Fig. 1.

#### X-ray diffraction (XRD)

Phase analysis was conducted for synthesized powdered samples using a Rigaku SE XRD machine, using a ceramic copper tube as the radiation source (Cu Kα, *λ* = 1.54060 Å). Data were recorded within a diffraction angle range of (2*θ* = 5–70°), with a standard operating temperature of 22–23 °C. The instrument was calibrated with standard silicon and compared with the standard ICDD database (card no. #: 01-074-0566). The operating voltage and current were maintained at 40 kV and 50 mA.

#### Scanning electron microscopy (SEM) analysis

Surface morphology was explored utilizing an FESEM machine (model: EVO18, Zeiss) and the images was captured maintaining 15 kV accelerating voltage.

#### UV-visible spectroscopic analysis

A UV-visible spectrometer (Hitachi U-2910) measured absorbance at wavelengths of 190 to 350 nm. A 1 cm width glass cell cuvette was used, and tungsten and deuterium lamps were used for UV-visible irradiation.

#### Photodegradation experiment

**Fig. 2:**
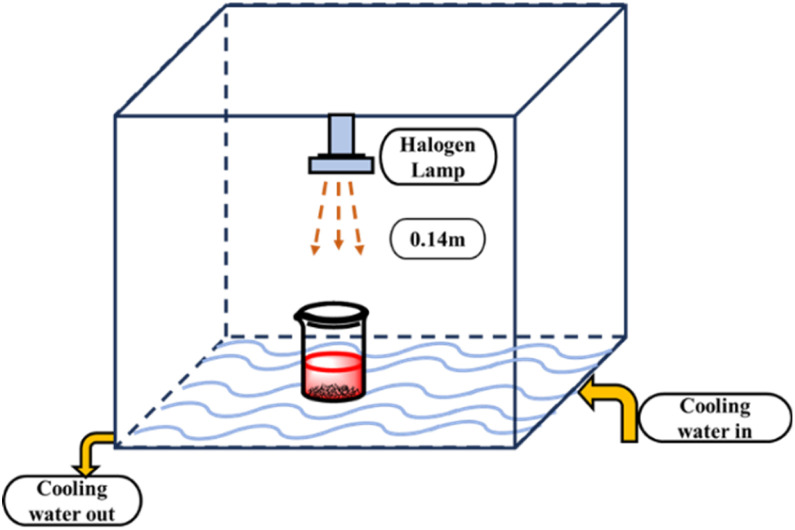
Experimental setup for photodegradation of HAp with simulated sunlight irradiation.

The photocatalytic activity of synthesized HAp was tested for the degradation of Congo red (CR) solution under simulated sunlight irradiation. A halogen lamp (SEN TAI JM-500) was mounted on a rigid wooden box wrapped with black cloth to prevent leakage. The box was placed over a cooling water bath, maintaining a temperature of approximately 25 °C with a continuous flow of cooling water. The distance between the lamp and the reaction mixture was 0.14 m, and no stirring was performed in both dark and irradiated experiments. The photo catalytic set-up is visualized in Fig. 2. The concentration of the dye solution was measured using a (Hitachi U-2910) UV-vis spectrophotometer.

## Results and discussion

### XRD data study

**Fig. 3:**
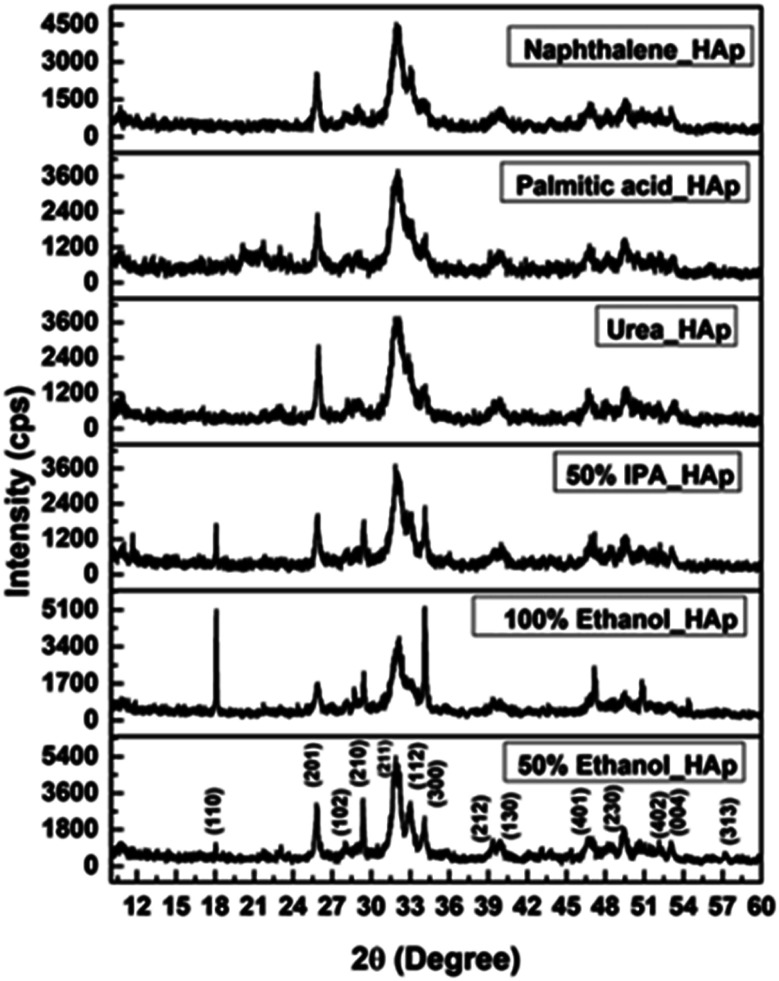
X-ray diffractogram of synthesized HAp using different solvent systems and organic modifiers.

The XRD patterns of the synthesized HAp using different solvent systems such as ethanol and IPA, as well as different organic modifiers, which include urea, palmitic acid, and naphthalene are displayed in Fig. 3, and the crystallographic parameters were studied from the generated patterns. The 2*θ* (degree) diffracted positions of the HAp phases were visualized at 18.09°(110), 25.93°(002), 31.83°(211), 32.24°(112), 32.96°(300), 34.12°(202), 39.88°(130), 46.75°(222) and 49.53°(213), which were matched with the standard ICDD database of the card no: #01-074-0565 for HAp and a hexagonal structure was predicted. very similar type of data were noticed for all the synthesized HAps. The observed results revealed that the synthesis media have a control on the crystallographic planes, *e.g.* in the case of the ethanol medium, the (110) plane exhibited significant intensity (which could potentially serve as a driving force for enhancing photocatalytic activity) whereas this plane was either absent or exhibited very weak intensity in other systems.

### Assessment of different crystalline parameters

The crystallographic analysis was performed here by computing relative intensity, preferential growth, lattice parameters, cell volume, crystallinity index, dislocation density, crystallite size, degree of crystallinity, and microstrain using various mathematical expressions, and the details were explained elsewhere.^3,31^

“Preferential growth” is considered one of the fundamental parameters, which is attributed to the “crystallite structure” from the XRD data.^34^ From the “relative intensities” of the corresponding XRD patterns, preferences were estimated. The relative intensity of the sample was estimated by implying the (211) plane alongside the ratio of the intensity of this plane to the intensities of the three peaks of the planes (002), (202), and (300). The mathematical expression for calculating “relative intensity (RI)” can be described as eqn (1).^14^

Relative intensity,1
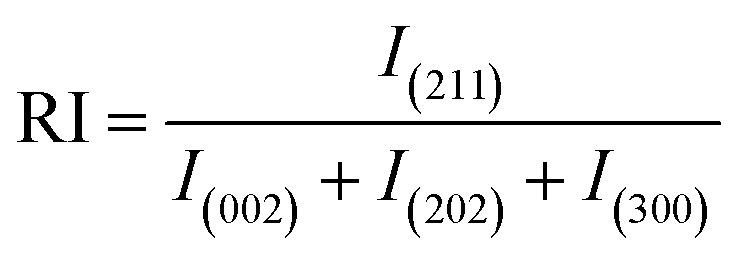
In the above-mentioned equation, the subscripts indicate the corresponding plane, whose intensity is to be calculated by considering the relative intensities of ICDD card no #01-074-0565. The measured relative intensity for ethanol HAp (50, 100% (v/v)), IPA_HAp (50% (v/v)), urea_HAp, palmitic acid_HAp, and naphthalene_HAp is listed in Table 1. The preference growth was calculated for synthesized HAp by utilizing mathematical expression (eqn (2)),^35^2
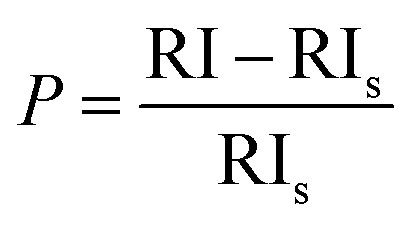
Here in eqn (2), the RI_s_ denotes the “standard relative intensity” of the identical plane. The estimated preferential growth for particular planes such as (202) and (002) show higher preferences as compared to the (211) plane. It's an indication of lower agglomeration of molecules on the surface of the specimen, which results in the dimensional stability of crystallites along with minimum surface energy.

**Table 1:** Preference growth and relative intensities of different HAp samples

Synthesized HAp from	Considered plane	The relative intensity of the sample	Standard relative intensity	Preferential growth
50% ethanol	002	0.3	0.19	0.578947
	211	0.56	0.848	−0.339622
	202	0.23	0.108	1.129629
	300	0.27	0.394	−0.314720
	110	0.03	0.012	1.13129
100% ethanol	002	0.18	0.19	−0.052631
	211	0.26	0.848	−0.693396
	202	1.4	0.108	11.96296
	300	0.053	0.394	−0.865482
	110	0.130	0.012	9.6863
50% isopropyl alcohol	002	0.35	0.19	0.842105
	211	0.56	0.848	−0.339622
	202	0.34	0.108	2.148148
	300	0.011	0.394	−0.972081
Urea	002	0.27	0.19	0.421052
	211	0.71	0.848	−0.162735
	202	0.18	0.108	0.666666
	300	0.26	0.394	−0.34010
Palmitic acid	002	0.37	0.19	0.947368
	211	0.74	0.848	−0.127358
	202	0.127	0.108	0.175925
	300	0.182	0.394	−0.538071
Naphthalene	002	0.52	0.19	1.736842
	211	0.8	0.848	−0.056603
	202	0.13	0.108	0.203703
	300	0.1	0.394	−0.746192

From the calculated preference growth, it was observed that the 50%_HAp sample was dominated by the growth of 110 and 202 planes and the rest of the planes were lower or negative. Relatively similar values were noticed for the 110 (1.129) and 202 (1.131) planes. Significant intensity was also visualized in the XRD figure for the 50% ethanol containing sample. On the other hand, when the percentage was increased from 50% to 100% of ethanol the preference growth of the 110 plane was significantly improved with a value of 9.6863. This was the most intense 110 plane among all the experimented samples. In tune with this plane, the 202 plane also exerted the highest preference growth for the 100% ethanol sample, and the value was 11.9629. The XRD figure also supported the finding of the strong peaks. The two planes (110 and 202) were preferred by the reaction conditions as well as the organic modifiers and solvent system as all the samples showed positive preference growth. Thus, it can be predicted that the organic modifiers and solvent system produce thermodynamically stable 110 and 202 planes for the hydroxyapatites. On the other hand, the standard database revealed that the 211 plane is the strongest plane for the synthesized hydroxyapatite. But, in this research, this plane was not favored by the reaction parameters and the modifiers as well as solvent system as all the samples exerted negative preference growth. Similarly, the thermodynamically unfavorable 300 plane was also found by the calculation of preference growth which carried negative values for all the samples.

The lattice parameters of crystallite HAp are indicated by *a*, *b*, and *c*; the planes of the unit cell are denoted as *h*, *k*, and *l* in eqn (3) and (4).

Lattice parameter equation,3
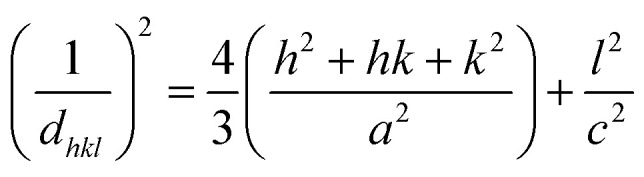


Cell volume,4
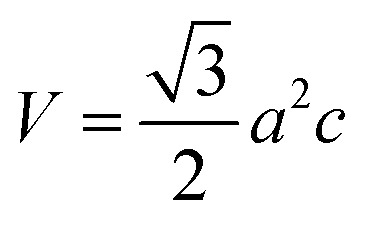
The measured values of the lattice parameter and cell volume are listed in Table 2. From the calculated data, it is prominently visible that the values match those of the standard database. The lattice parameter, as well as the volume of unit cells for synthesized HAp, varies for different solvent systems. However, the highest values of lattice parameters and cell volume were obtained for HAp, which was synthesized from organic modifiers such as urea, palmitic acid, and naphthalene.

**Table 2:** Crystallographic parameters of the prepared HAp

Parameter	50% ethanol_HAp	100% ethanol_HAp	50% IPA_HAp	Urea_HAp	Palmitic acid_HAp	Naphthalene_HAp
Lattice parameter	*a* = *b*= 9.38	*a* = *b*= 9.38	*a* = *b* = 9.35	*a* = *b* = 9.70	*a* = *b* = 9.65	*a* = *b*= 9.68
	*c* = 6.9	*c* = 6.88	*c* = 6.88	*c* = 6.86	*c* = 6.86	*c* = 6.90
The volume of the unit cell, *V* (Å^3^)	525.75	524.23	520.88	558.98	553.23	559.92
Crystallite size, *D*_c_ (nm)	26.16	51.18	24.72	11.50	11.59	9.06
Degree of crystallinity, *X*_c_	0.0381	0.030	0.020	0.0038	0.020	0.012
Microstrain, *ε*	0.0108	0.0117	0.0132	0.0234	0.0134	0.0157
Dislocation density, *δ* (10^15^ lines per m^2^)	1.46	0.38	1.63	7.56	7.44	12.18
Crystallinity index, CI_XRD_	0.81508	2.73958	0.86976	0.2517	0.4556	0.312744
Specific surface area, *S* (g^−1^ m^2^)	0.072	0.037	0.076	0.165	0.163	0.20

In multidisciplinary applications, crystal dimensions play a critical role. Small crystals are often distinguished by maximum surface area or *vice versa*.^36^ Furthermore, the physical as well as chemical properties of materials can vary based on crystal size. In X-ray diffraction and crystallography, the Scherrer equation relates the size of sub-micrometer crystallites in a solid to the widening of a diffraction peak, which estimates powder crystal size using eqn (5).^24,37^ The equations focus on crystallite size, not particle size, as particles are often agglomerations of multiple crystallites. XRD does not provide information about particle size, but techniques such as visible light scattering, image analysis, and sieving can be used for direct measurement.^38–40^

Crystallite size,5
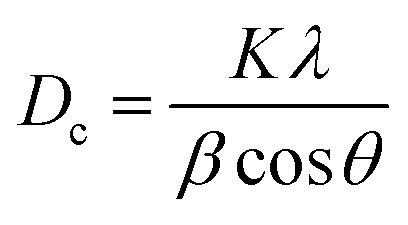
In the above-mentioned equation, *K* = shape factor (arbitrary constant), whose magnitude is 0.94; *D*_c_ = crystallite size (nanometer or angstrom), *θ* = diffraction angle (in degree), and *β* = FWHM (full width at half maximum) in radian. The analysis of crystal size from different solvent systems and numerous organic modifiers shows a significant variation. It is prominently visible that, while using an organic modifier, the size of the repeating unit decreased. Conversely, the maximum crystallite size is obtained from ethanol HAp (100% (v/v)), while the lowest dimensions are shown in HAp when naphthalene is used.

The orientation of an atom or molecule in three-dimensional space is explained by the term known as degree of crystallinity. Although the behavior of materials is influenced by crystallinity, regulating the degree of crystallinity is very challenging.^31^ The degree of crystallinity is expressed in eqn (6), and the calculated data are listed in Table 2. The minimum levels of crystallinity are obtained for HAp, which is synthesized utilizing urea, while the maximum degree of crystallinity is obtained for HAp synthesized from 50% (v/v) ethanol.

Degree of crystallinity,6
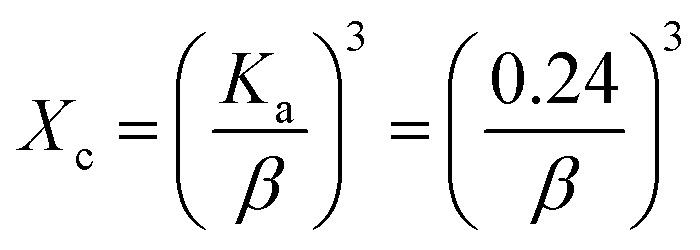


Microstrain in crystalline substances pertains to the internal stresses of crystal planes, which may be compressive or tensile. This stress induces crystallite deformation, affecting the characteristics and application of the materials.^31^ The mathematical expression for calculating microstrain is shown in eqn (7), and measured values are shown in Table 2.

7 *ε* = *β*/4 tan *θ*

From the estimated values, it's visible that utilizing organic modifiers such as urea, palmitic acid, and naphthalene results in a high amount of microstrain.

In crystalline materials, defects, often as dislocation, are generally composed of imperfections, including line, point, and area dislocation. Line dislocation was described, and values were generated using eqn (8), and data are shown in Table 2.

Dislocation density,8
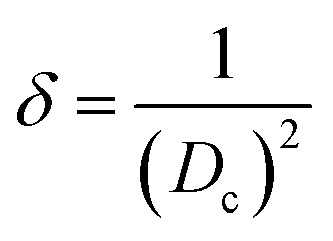


A significant change in dislocation density was observed for HAp, which was synthesized by employing an organic modifier. Conversely, minimal values are obtained when using different solvent systems.

Crystallinity quantifies the crystalline component of materials, characterizing crystal perfection, range, and size. The crystallinity index (CI) is quantitatively evaluated using XRD data by applying eqn (9). The calculated values are illustrated in Table 2.

Crystallinity index,9
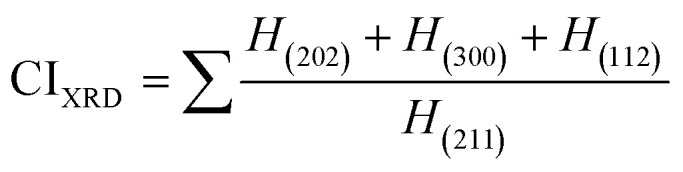


The maximum crystalline index was observed for 100% ethanol_HAp, while the lowest value was found for 50% ethanol_HAp.

The specific surface area, a critical characteristic in HAp characterization and application, is predominantly connected to crystallite size and was computed using eqn (10) and is presented in Table 2.

Specific surface area,10
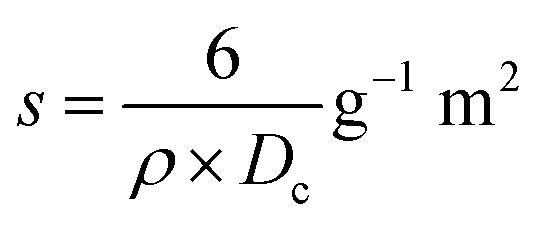


Specific surface area is the reciprocal term of crystal size. In the above-mentioned equation, crystallite size and density of HAp were represented by *D*_c_ and *ρ* (3.16 g cm^−3^). In this experiment, higher surface area (0.20 g^−1^ m^2^) is obtained when naphthalene is used as a modifier.

### Crystallite size estimation by employing various models

#### Liner straight-line method of Scherrer's equation

**Fig. 4:**
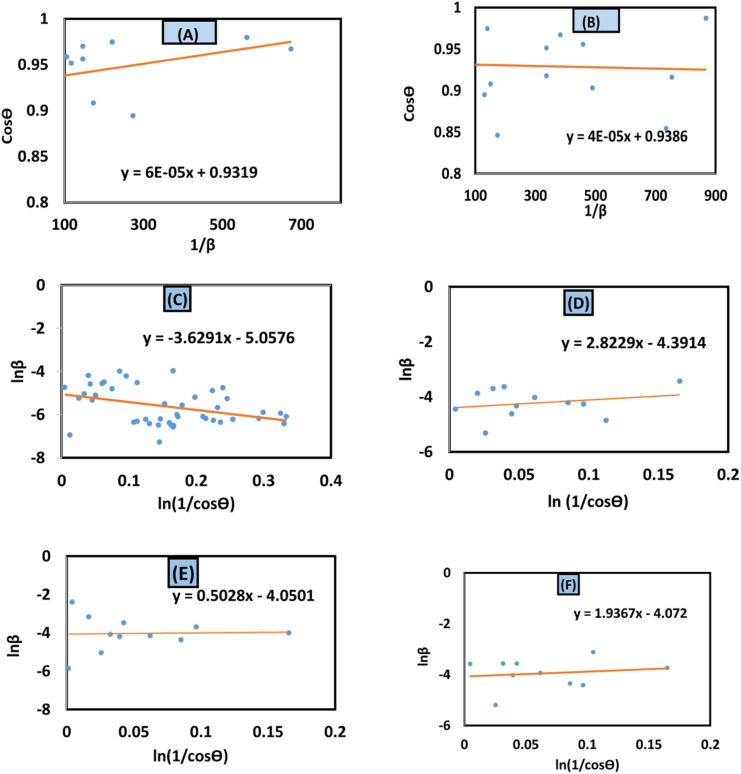
Linear straight-line model of Scherrer's equation for crystallite size estimation for (A) 50% ethanol_HAp, (B) 100% ethanol_HAp, (C) 50% IPA_HAp, (D) urea_HAp, (E) palmitic acid and (F) naphthalene_HAp.

The dimensions of the unit cell can be determined by applying the straight-line method of Scherrer's equation, which is the result of different arrangements of Scherrer's equation shown in eqn (5). The mathematical expression of this method can be rearranged as eqn (11).^37^11
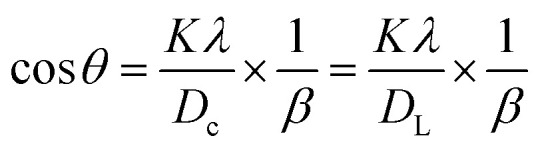
In this equation, crystallite size is denoted as *D*_c_. To obtain XRD data from the above-mentioned equation, this equation is correlated with (*y* = *mx* + *c*). To present this equation in a graph, cos *θ* and 1/*β* are plotted on the *y*-axis and *x*-axis (Fig. 4(A)–(F)), respectively. From this model, the highest, as well as lowest, crystallite size was obtained from 100% ethanol_HAp (3466 nm) and naphthalene_HAp (700 nm). Additionally, introducing organic modifiers such as urea, palmitic acid, and naphthalene results in the lowest crystallite size.

#### Monshi–Scherrer model

The Monshi–Scherrer model, the logarithmic form of the Scherrer equation, was utilized to investigate the size of the crystallite of synthesized HAp further.^1^ The mathematical expression of this model is shown in eqn (12).12
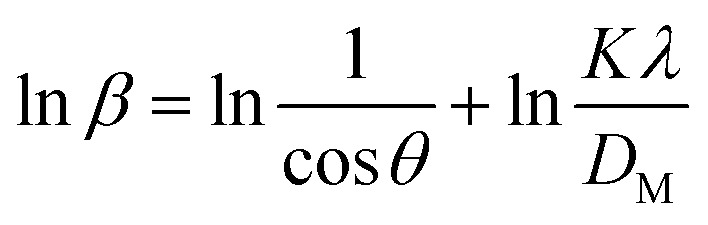


**Fig. 5:**
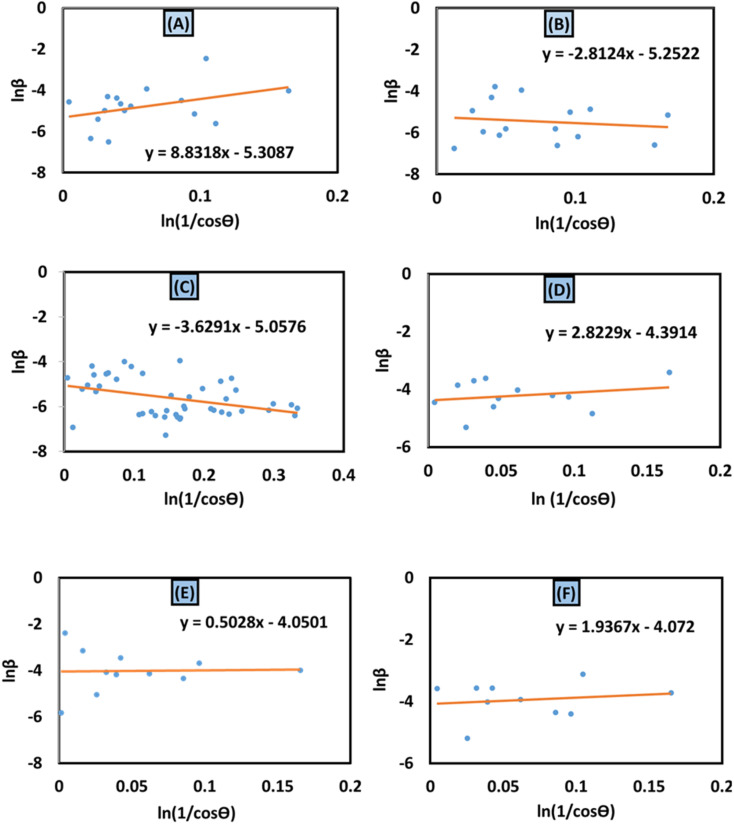
Monshi–Scherrer's model for calculating crystallite size for (A) 50% ethanol_HAp, (B) 100% ethanol_HAp, (C) 50% IPA_HAp, (D) urea_HAp, (E) palmitic acid_HAp and (F) naphthalene_HAp.

To determine the crystallite size from this equation, ln(1/cos *θ*) in (*x*-axis) and ln *β* in (*y*-axis) were potted (Fig. 5(A)–(F)), which was then compared with a straight line equation, resulting in the following equations.13
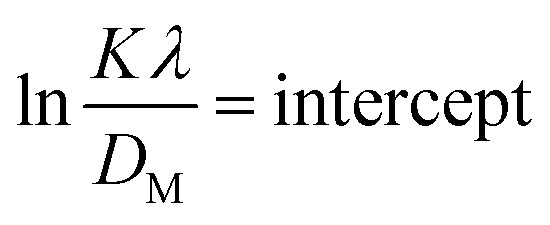
or,14
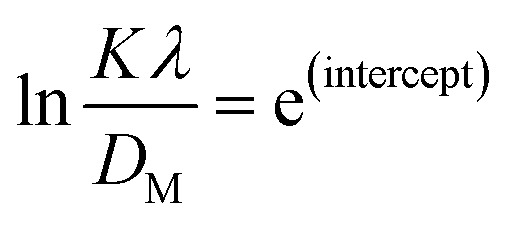


The crystallite size was calculated from the plot, and the results are shown in Table 3. The measured size of the unit cells is 28.02 nm for 50% ethanol_HAp, 26.48 nm for 100% ethanol_HAp, 21.79 nm for 50% IPA_HAp, 11.19 nm for urea_HAp, 7.95 nm for palmitic acid_HAp, and 8.13 nm for naphthalene_HAp.

**Table 3:** Microstructural characteristics of hydroxyapatite utilizing various models in this study

Model name		Crystal size (in nm), stress (in N m^−2^), and energy density (in J m^−3^)					
		50% ethanol	100% ethanol	50% IPA	Urea	Palmitic acid	Naphthalene
The linear straight-line method of Scherrer's equation (*D*_L_)		2310.9	3466.35	1980.77	806.12	936.85	700.27
Monshi–Scherrer's method (*D*_M_)		28.0202	26.480	21.7981	11.1967	7.9591	8.13541
Williamson–Hall method	UDM	*ε* = 0.0036	*ε* = −0.0028	*ε* = 0.0064	*ε* = 0.0004	*ε* = −0.016	*ε* = −0.0003
		*D*_w_ = 32.24	*D*_w_ = 13.59	*D*_w_ = 106.66	*D*_w_ = 106.65	*D*_w_ = 3.2778	*D*_w_ = 6.5712
	USDM	*σ* = 22 639	*σ* = −16885	*σ* = 38 378	*σ* = 13 967	*σ* = −95990	*σ* = 7 × 10^10^
		*D*_w_ = 49.51	*D*_w_ = 13.593	*D*_w_ = 106.65	*D*_w_ = 11.554	*D*_w_ = 3.277	*D*_w_ = 1.17 × 10^−5^
	UDEDM	*u* = 5.287	*u* = −4.87	*u* = 11.079	*u* = 4.0319	*u* = −11 × 10^11^	*u* = 2 × 10^7^
		*D*_w_ = 30.81	*D*_w_ = 13.59	*D*_w_ = 106.65	*D*_w_ = 10.917	*D*_w_ = 32.7787	*D*_w_ = 1.17 × 10^−6^
The size–strain plot (*D*_w_)		8.7203	7.0380	15.57910	10.748	1.4845	173.3175
Halder–Wagner method, (*D*_w_)		1.91570	7.2463	8.0645	7.8740	55.555	3.448
Sahadat–Scherrer's model (*D*_w_)		51.353	81.561	69.327	15.406	25.6766	13.4615

#### Williamson–Hall method

The Scherrer equation primarily analyzed crystallite size in XRD patterns, disregarding lattice conditions influenced by defects, faults, grain boundaries, and lattice strain.^41,42^ However, the Williamson–Hall (W–H) and Warren–Averbach methods calculate intrinsic strain and particle size based on strain-induced XRD peak broadening. The W–H method is a simplified approach that considers the physical line broadening of the X-ray diffraction peak due to nanocrystal size and microstrain, resulting in a total broadening of the X-ray diffraction peak.^43,44^ The sum of XRD pattern widening can be expressed as

15 *β*_total_ = *β*_size_ + *β*_strain_

The lattice strain of synthesized HAp, as well as crystallite size, was estimated using several modified W–H analyses, which include the uniform deformation model (UDM), uniform stress deformation model (USDM), and uniform deformation energy density model (UDEDM).

#### Uniform deformation model (UDM)

The UDM considers homogeneous crystal strain in all planes, making lattice strain an isotropic feature. The physical peak widening produced by lattice strain can be represented as:^45^

16 *β*_strain_ = 4*ε* tan *θ*

The measured peak widening for a peak with a *hkl* (*β*_*hkl*_) quantity can potentially be estimated by adding the size of the crystallite and strain contributions, leading to a Cauchy-like profile. From eqn (5) and (16), the total line breadth can be17
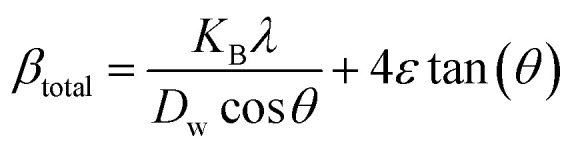
Rearranging eqn (17) forms18
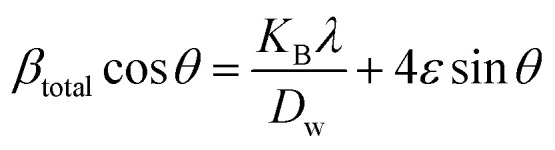


**Fig. 6:**
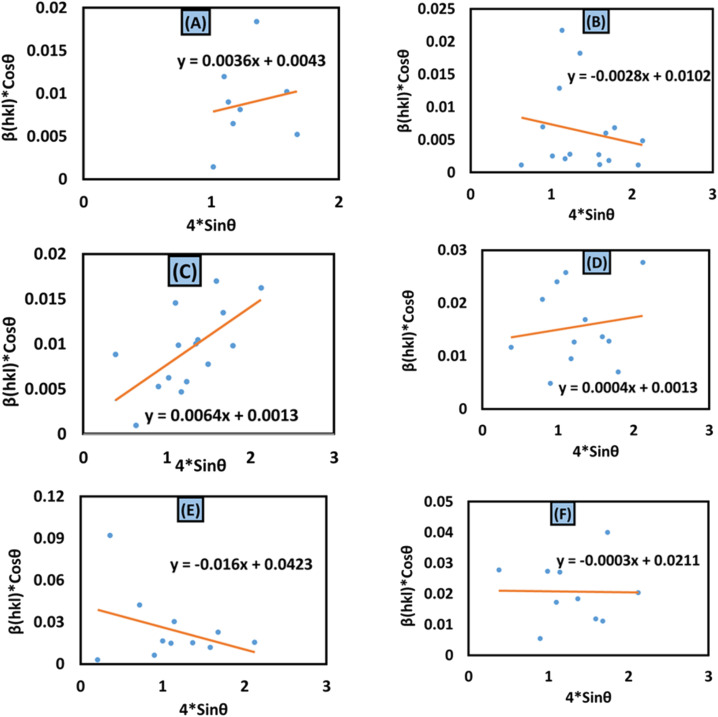
Uniform deformation model for calculating crystallite size for (A) 50% ethanol_HAp, (B) 100% ethanol_HAp, (C) 50% IPA_HAp, (D) urea_HAp, (E) palmitic acid_HAp and (F) naphthalene_HAp.

The aforementioned mathematical expression is the linear plot for UDM. A straight line is observed while plotting *β*_*hkl*_ cos *θ* (in the *y*-axis) and 4*ε* sin *θ* (in the *x*-axis) (Fig. 6(A)–(F)); the slope of this equation corresponds to the lattice strain, and the intercept indicates crystallite dimensions. The resultant crystallite size and microstrain were calculated and are listed in Table 3. The maximum crystallite size, as well as tensile deformation, was observed for 50% IPA_HAp and urea_HAp. Conversely, the rest of the synthesized HAps show compressive deformation and comparatively small unit cells.

#### Uniform stress deformation model (USDM)

The real crystals are often anisotropic in nature. For this reason, the assumption on which the UDM is built isn't entirely justified. Therefore, the term incorporating the anisotropic nature is necessary to estimate lattice strain in the W–H model. In the uniform stress deformation model (USDM), stress due to lattice deformation, along with a small amount of microstrain in all crystallographic directions, is uniformly considered.^45^ From Hooke's law,

19 *σ* = *εY*_*hkl*_

**Fig. 7:**
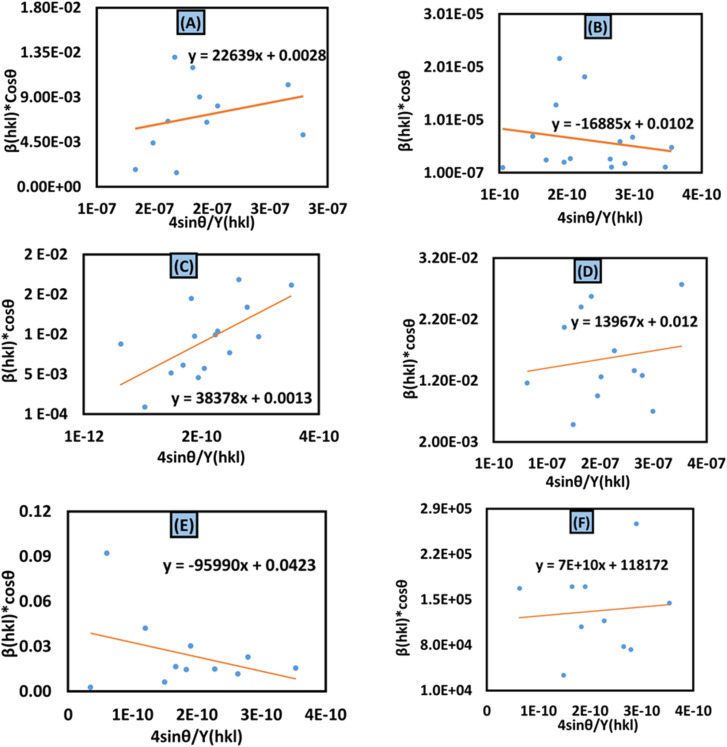
Uniform stress deformation model for calculating crystallite size for (A) 50% ethanol_HAp, (B) 100% ethanol_HAp, (C) 50% IPA_HAp, (D) urea_HAp, (E) palmitic acid_HAp and (F) naphthalene_HAp.

From eqn (18) and (19), the amended form of the W–H model can be written as20
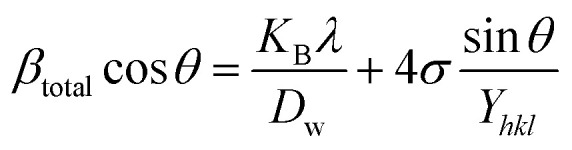
In eqn (20), *Y*_*hkl*_ indicates the modulus of elasticity, which is perpendicular to the crystallographic planes.^46^ Thus, plotting *β*_total_ cos *θ* along the *y*-axis and 4 sin *θ*/*Y*_(*hkl*)_ along the *x*-axis produces a linear graph (Fig. 7(A)–(F)). The gradient of this expressed straight line delivers a measure of stress (*σ*), whilst the point of intersection offers the crystallite size *D*_w_ of the HAp nanocrystals. The crystal structure of synthesized HAp nanoparticles, as well as the stress obtained using this model, is listed and shown in Table 3. From the analysis, it is prominently visible that the largest crystalline HAp (106.65 nm) is produced while using 50 volume % IPA as a medium.

#### Uniform deformation energy density model (UDEDM)

In consideration of real crystals, the assumption of linear proportionality and crystal isotropy between strain and stress cannot be considered because of numerous defects, dislocation, and agglomerates, which create imperfections in all types of crystalline material.^47^ Therefore, a perfect model is necessary, which should be useful for further study. The energy density (*u*) is obtained from Hooke's law, which is21
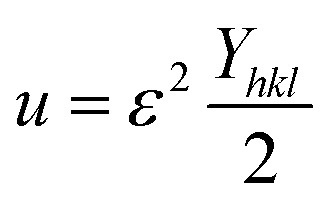


Altering eqn (21) in terms of *ε* and substituting this value into eqn (17), the UDEDM equation is obtained as follows:22
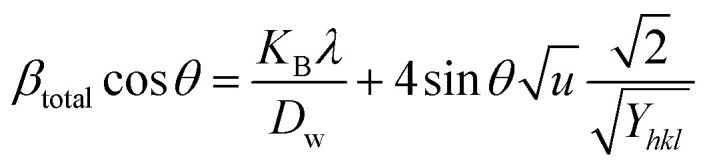


**Fig. 8:**
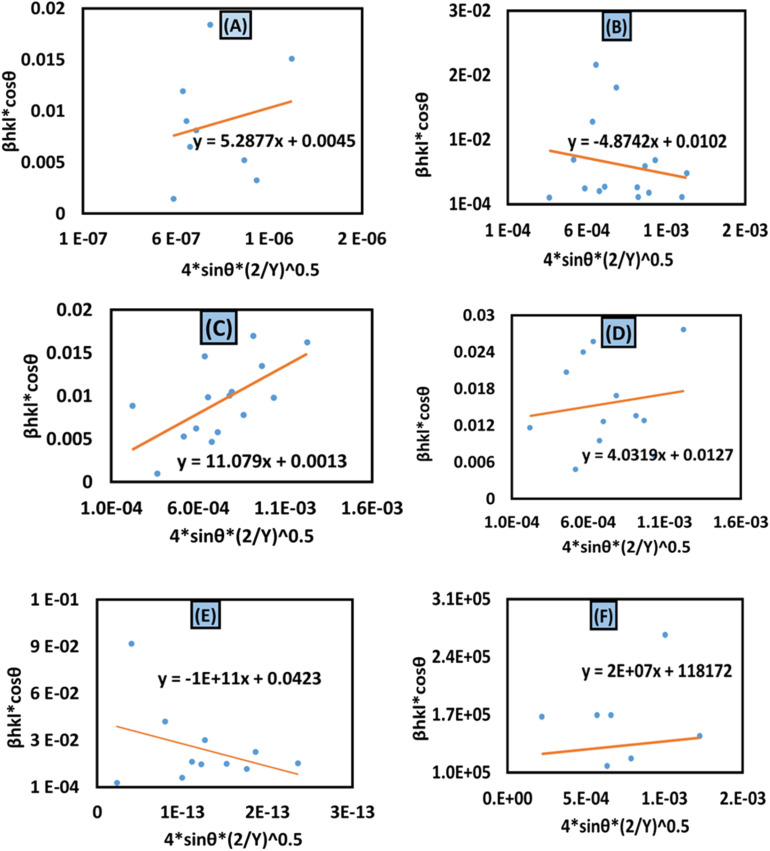
Uniform deformation energy density model for calculating crystallite size for (A) 50% ethanol_HAp, (B) 100% ethanol_HAp, (C) 50% IPA__HAp, (D) urea_HAp, (E) palmitic acid_HAp and (F) naphthalene_HAp.

The calculated crystallite size from this model is shown in Table 3. The highest crystalline HAp particle is obtained from using 50% IPA as a solvent system. Conversely, the lowest unit cell is obtained by using naphthalene as an organic modifier (Fig. 8).

#### Size–strain plot (SSP) method

In XRD crystallographic analysis, crystallite dimensions, as well as lattice strain, are considered important factors in evaluating peak widening. The size–strain plot (SSP) method is utilized to distinguish between these two factors, which not only gives a better understanding of strain-size parameters but also an isotropic essence of the crystal structure.^48^ In this model, the extent of the crystallite size and strain is identified as a Lorentzian as well as a Gaussian function, respectively.^49,50^ Mathematically, this expression can be written as

23 *β*_total_ = *β*_L_ + *β*_G_

**Fig. 9:**
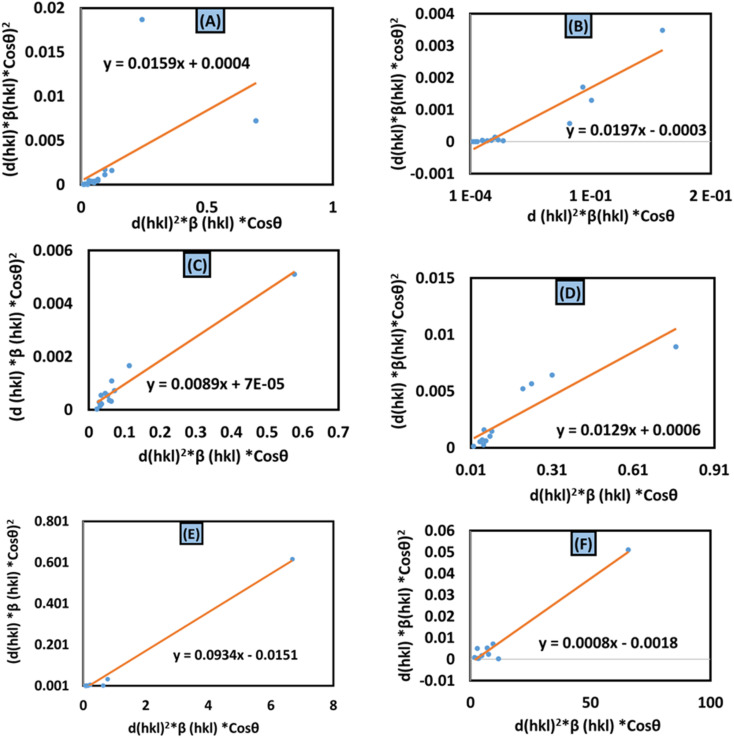
Size–strain plot method for calculating crystallite size for (A) 50% ethanol_HAp, (B) 100% ethanol_HAp, (C) 50% IPA_HAp, (D) urea_HAp, (E) palmitic acid_HAp and (F) naphthalene_HAp.

24
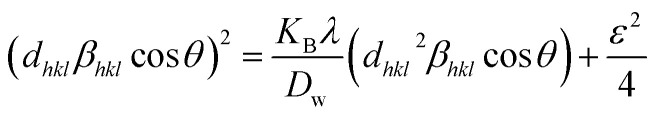
where the distance between lattice planes (*hkl*) is denoted by *d*_*hkl*_. By plotting (*d*_*hkl*_*β*_*hkl*_ cos *θ*)^2^ and (*d*_*hkl*_^2^*β*_*hkl*_ cos *θ*) in the *x*-axis and *y*-axis (Fig. 9(A)–(F)), the strain and size can be calculated by assessing the *y*-intercept and slope of the linear fit correspondingly.

According to this model, the crystalline HAp produced is listed in Table 3. This analysis shows that a large crystal size (173 nm) was obtained while using naphthalene as an organic modifier. On the other hand, the lowest crystalline HAp (1.48 nm) was produced by utilizing palmitic acid as an organic modifier.

#### Halder–Wagner method

The Halder–Wagner technique implies that XRD peak widening is a symmetric Voigt function, solving the difficulties raised by the SSP method. The XRD peak suits the Gaussian function. However, the tail areas don't. The technique entails the symmetric Voigt function as a convolution of both Lorentzian and Gaussian functions.^45^ The whole extent of a Voigt function, as defined by Halder and Wagner, is represented as:

25 *β*_*hkl*_^2^ = *β*_L_*β*_*hkl*_ + *β*_G_^2^

**Fig. 10:**
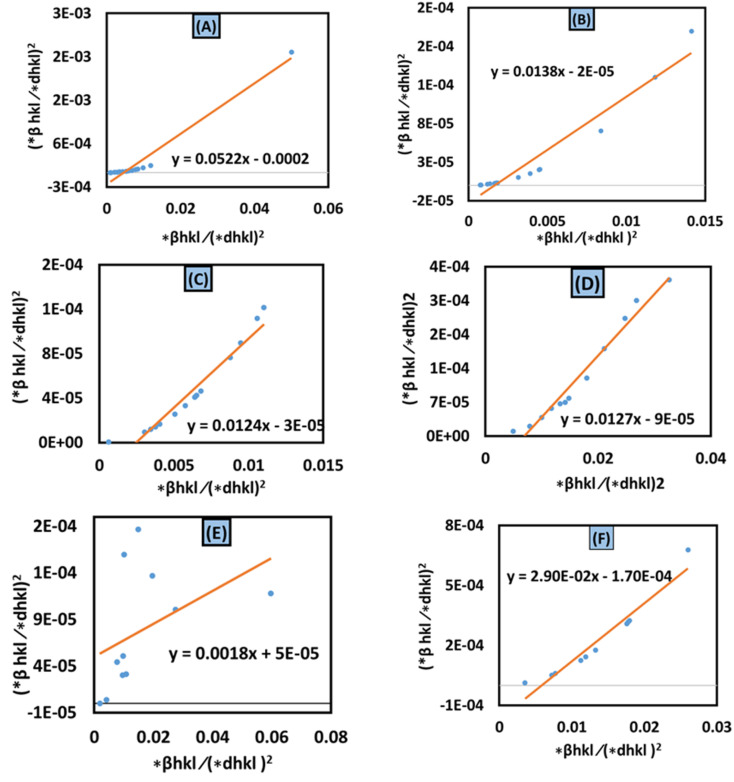
Halder–Wagner method for calculating crystallite size for (A) 50% ethanol_HAp, (B) 100% ethanol_HAp, (C) 50% IPA_HAp, (D) urea_HAp, (E) palmitic acid_HAp and (F) naphthalene_HAp.

In the above-mentioned equation, the full width at half maximum of the Gaussian function and the Lorentzian function is denoted by *β*_G_ and *β*_L_. According to this model, the lattice strain and crystallite dimensions can be written as26
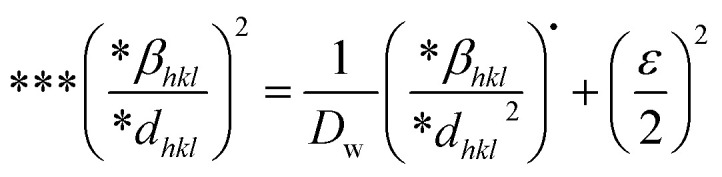
where 
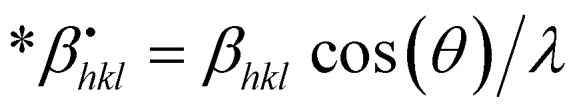
 and 
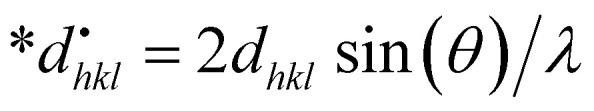
. By plotting eqn (26) with (*β_*hkl*_/*d_*hkl*_)^2^ within the *y*-axis and (*β_*hkl*_/*d_*hkl*_2) term in the *x*-axis (Fig. 10(A)–(F)), the size of crystallite material along with the intrinsic strain of the HAp nanomaterial is calculated. From this model, the estimated size of the synthesized HAp is shown in Table 3. The largest size of the nanocrystalline HAp (55.55 nm) is produced when using palmitic acid as an organic modifier. Conversely, small crystalline HAp (1.915 nm) was observed while employing 50% ethanol as a medium.

#### Sahadat–Scherrer model

The Sahadat–Scherrer model is an approach employed to determine the crystal size of substances by eliminating a certain number of the limits related to existing models and delivering a more exact evaluation.^51^ The concept involves creating a straight line that follows the point of origin, leading to a more precise calculation of the dimensions of the crystallite.^52^ The mathematical expression of this model can be written as eqn (27).27
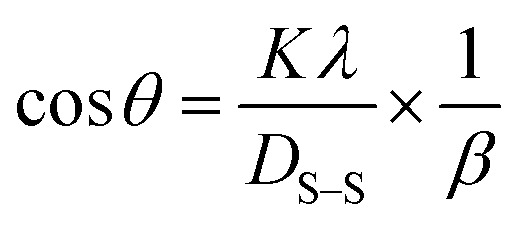


**Fig. 11:**
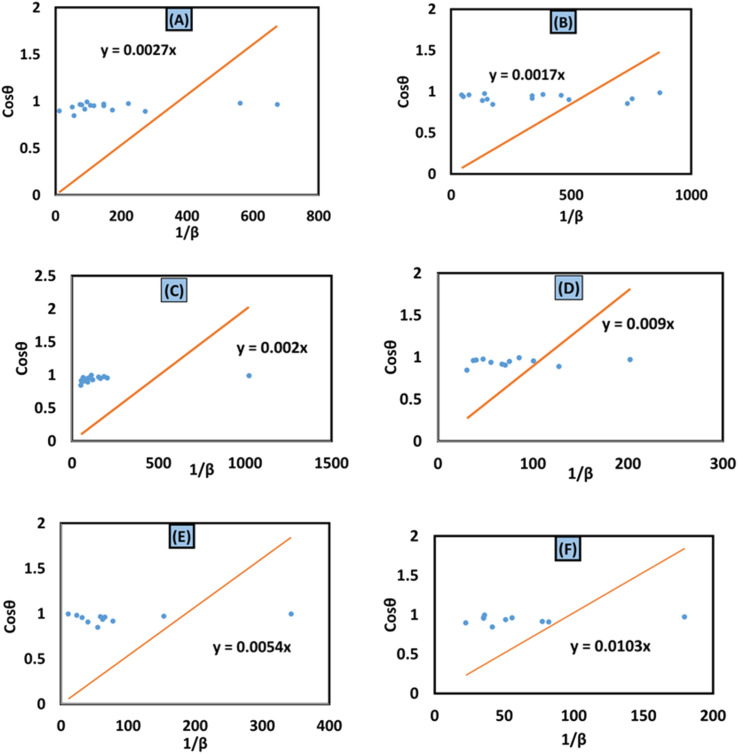
Sahadat–Scherrer model for calculating crystallite size for (A) 50% ethanol_HAp, (B) 100% ethanol_HAp, (C) 50% IPA_HAp, (D) urea_HAp, (E) palmitic acid_HAp and (F) naphthalene_HAp.

To plot a graph using this model, 1/*β* on the *x*-axis and cos *θ* on the *y*-axis were built (Fig. 11(A)–(F)). An intercept going through the origin was drawn, generating a straight line and it was compared with the *y* = *mx* equation to compute crystallite size. The calculated size of crystalline HAp is shown in Table 3. According to this model, the highest crystalline HAp (51 nm) was obtained while using 50% ethanol as a solvent.

#### Scanning electron microscopy (SEM)

**Fig. 12:**
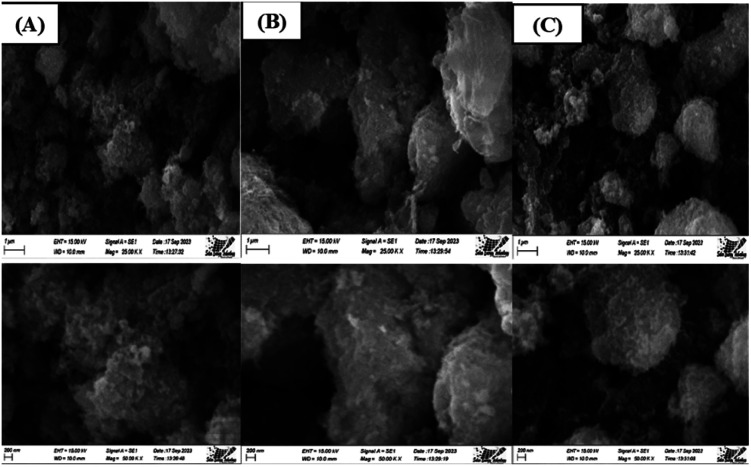
Scanning electron micrograph of Synthesized HAp using different modifiers, (A) urea, (B) palmitic acid, and (C) naphthalene.

The SEM images of synthesized HAp are shown in (Fig. 12(A)–(C)), where different types of organic modifiers (urea, palmitic acid, and naphthalene) are used. Organic modifiers play a critical role in changing the crystal structure as well as its morphology. From analyzing the images, it's prominently visible that many spherical nanoparticles were present in the synthesized HAp, which tend to agglomerate. Apart from this, most of these particles were less than a hundred nanometers, as a prominent form. Conversely, we discovered several associated particles with no distinct geometric form.

#### Photocatalytic activity

UV-vis spectrophotometry was used to measure the amount of photodegradation of synthesized HAp. The degradation percentage (*D*_p_) and degradation capacity (*q*_e_) were calculated by utilizing eqn (27) and (28).

Degradation percentage:28
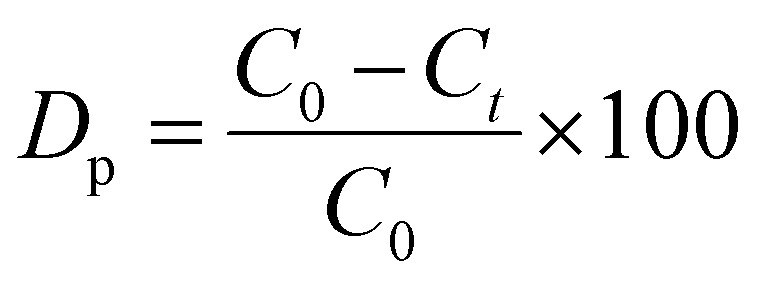


Degradation capacity:29
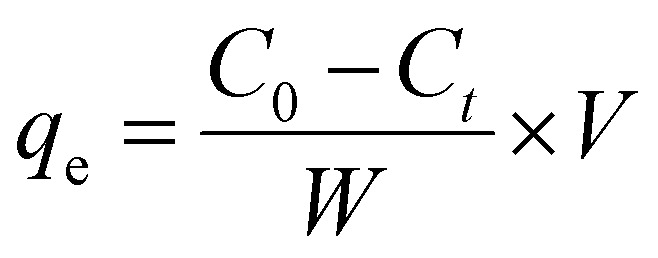
In the above-mentioned equation, *C*_*t*_ and *C*_0_ denote the final as well as initial concentration of the samples at the time “*t*” correspondingly.^53^ Meanwhile, rast, *V* and *W* represent the volume of an aqueous solution of dye and the weight of the catalyst, respectively.

#### Photocatalytic activity of synthesized HAp

##### Effect of irradiation time

**Fig. 13:**
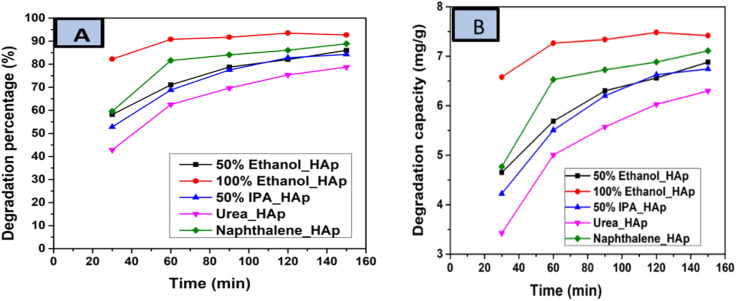
(A) Degradation percentage and (B) degradation capacity of synthesized HAp in terms of several times for 0.05 g of catalyst, 40 mL dye solution, and 90 min time.

The photodegradation of Congo red dye increased with time. In this experiment, up to 150 min, the calculated degradation percentage of the synthesized HAp using different solvent systems as well as different organic modifiers shows the lowest value for urea_HAp (69.63%) and the highest value for 100% ethanol_HAp (91.79%). Conversely, the minimum degradation capacity of synthesized HAp was observed from urea_HAp (5.57 mg g^−1^), and the maximum was investigated for 100% ethanol_HAp (7.33%). Catalyst amounts of 0.05 g and 90 min were precisely chosen for additional exploration. The degradation percentage, as well as the degradation capacity, is illustrated in (Fig. 13(A) and (B)).

##### Effect of catalyst dose

**Fig. 14:**
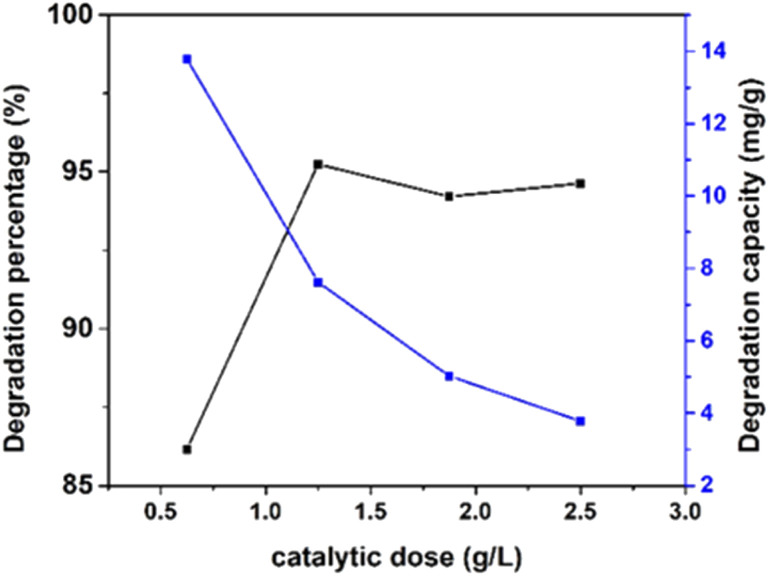
Effect of various dosages (0.625, 1.25, 1.875, and 2.5 g L^−1^) of the photocatalyst with fixed 10 ppm of 40 mL Congo red dye, 0.05 g of catalyst, and 90 min time on 100% ethanol_HAp.

Adsorbent capacity in a batch-operated system is substantially impacted by the adsorbent/solution ratio, which considerably influences its photocatalytic activity.^54^ The degradation percentage, as well as degradation capacity, was evaluated by altering the photocatalyst dose from 0.6 g L^−1^ to 2.5 g L^−1^ of 100% ethanol_HAp, which follows the above-mentioned experimental conditions, and the result is described in Fig. 14. The degradation percentage increased, with the increment in the catalytic dose, where the lowest (86%) and the highest (95%) values were obtained for synthesized HAp by using 100% ethanol as a solvent system. This phenomenon can be attributed to the fact that the higher the catalyst dosage, the more hydroxyl radicals can be generated due to the activation of more semiconductor molecules. However, this can reduce photon penetration by the aggregated solid and increase the scattering of photons, leading to fewer OH radicals and ultimately reducing photodegradation performance.^9^

Conversely, the degradation capacity abated with the increment in the catalytic dose, where the minimum (3%) and the maximum (13%) values were calculated for 100% ethanol_HAp correspondingly.

##### Effect of pollutant concentration

**Fig. 15:**
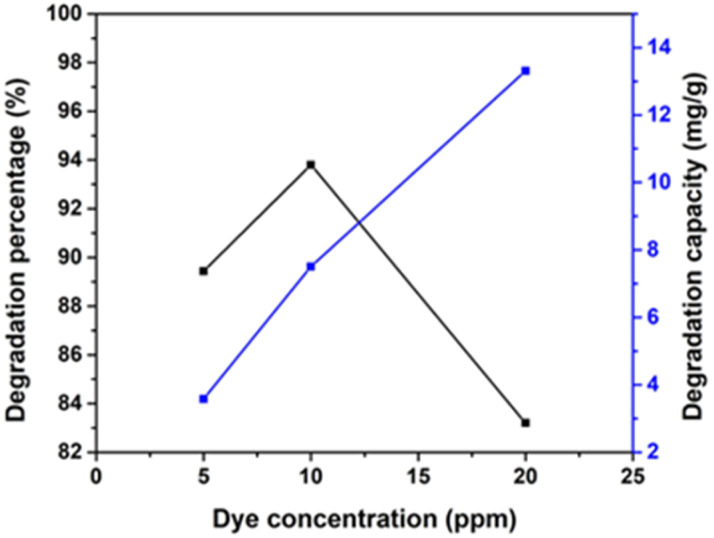
Impact of various initial dye concentrations (5, 10, and 20 ppm) for synthesized HAp using 100% ethanol as a medium for 0.05 g of catalyst, 40 mL dye solution, and 90 min time.

Substrate concentration is a crucial factor that ensures the efficient degradation of a pollutant.^55^ The study reveals that the concentration of CR, ranging from 5 to 20 ppm, significantly impacts its photocatalytic degradation, as depicted in Fig. 15. The increase in the dye concentration leads to a significant increase in degradation efficiency. This is due to the short lifetime of hydroxyl radicals, which can only react where they are formed. The higher the dye concentration, the greater the collision probability between organic matter and oxidizing species. The degradation efficiency decreases with an increase in the initial concentration of the dye from 10 ppm to 20 ppm, which is 93.8% to 83.2%. The degradation percentage decreases with an increasing dye concentration due to two reasons. First, more dye molecules are adsorbed on the photocatalyst surface, reducing the active sites and the generation of hydroxyl radicals. Second, the number of photons arriving on the catalyst's surface decreases as more light is absorbed by dye molecules, reducing the excitation of photocatalyst particles by photons. This results in wasting more incident light and increased radical precursor excitation.^56,57^ As the catalyst dosage increases, the availability of more active sites for catalysis increases, resulting in a greater degradation rate. Beyond the appropriate dosage, adding an additional catalyst may lead to extensive generation of active sites, which can restrict reactant accessibility and impair the overall effectiveness of the catalytic process. Gradually raising the catalyst dose beyond the first optimal range might produce additional active sites or strengthen certain catalytic properties, thereby increasing the degradation percentage.

##### Optical properties

The macroscopic optical futures of samples can be studied through absorption data or internal emitted light loss, relating diffused reflected photons to the samples' optical properties, particle size dispersion, and filling factor.^58,59^ The absorption frequency of synthesized HAp is used to calculate the optical band gap in the UV-vis spectrophotometer. For direct band gap analysis, the Tauc plot method was used.^2,60^ The mathematical expression of this method is shown in eqn (30).

30 *αhθ* = *A*(*hθ* − *E*_g_)^*n*^

**Fig. 16:**
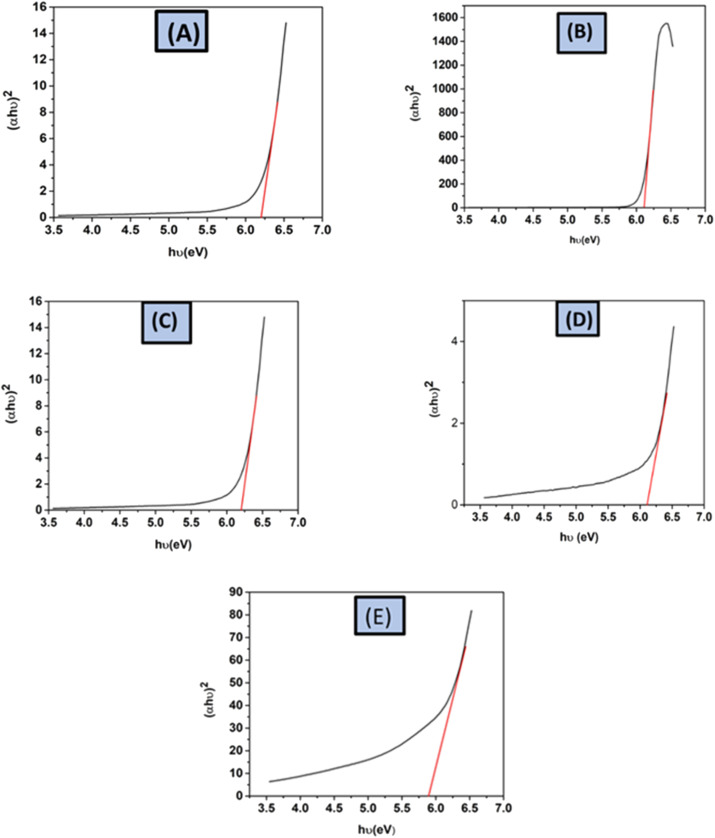
Optical band gap of synthesized HAp from different solvent systems, (A) 50% ethanol_HAp, (B) 100% ethanol_HAp, (C) 50% IPA_HAp, (D) urea_HAp and (E) naphthalene_HAp.

The equation uses Planck's constant (*h* = 6.626 × 10^−34^ J s) and light frequency (*θ*), respectively. Beer–Lambert law defines ‘*α*’ as the absorption coefficient, which is related to the absorbance of the sample (*A*) and the sample thickness (*d*) (*α* = ([2.303 × *A*]/*d*)). The allowed indirect as well as direct electronic transitions correspond to ‘*n*’ values of 2 and 1/2, whereas the forbidden direct and indirect transitions correspond to values of 3/2 and 3.^59,61,62^ The optical band gaps of the synthesized HAp were calculated (6.19 eV) for 50% ethanol_HAp, (6.11 eV) for 100% ethanol_HAp, (6.17 eV) for 50% IPA_HAp, (6.17 eV) for urea_HAp, and (5.89 eV) for naphthalene_HAp, correspondingly shown in (Fig. 16(A)–(E))

Semiconducting-based heterogeneous photocatalysis involves the production of electron–hole pairs when a semiconductor is irradiated with a photon of energy equal to or greater than its band gap width. These pairs can migrate to the catalyst surface, participating in redox reactions with adsorbed pollutant molecules and degrading them into smaller fragments. Another strategy involves reaching holes on the catalyst's surface reacting with surface-bonded H_2_O or OH^−^ to produce hydroxyl radicals and electrons reacting with adsorbed dissolved oxygen to produce superoxide radicals. These radicals attack organic pollutants and degrade them into smaller fragments. Further attacks on these radicals can mineralize the produced intermediates into water, carbon dioxide, and other inorganic species if nitrogen or sulfur-containing compounds are present.^63,64^ The degradation of Congo red by employing HAp was proposed and is shown in eqn (31)–(35).31
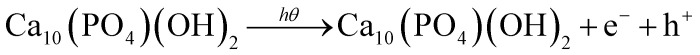

32 h^+^ + e^−^ + H_2_O → ˙OH^−^ + ˙O_2_ + ˙O_2_^−^ + OH_˙_^−^ + H^+^

33 ˙OH_˙_^−^ + ˙OH_˙_^−^ → H_2_O_2_

34 H_2_O_2_ + e^−^ + h^+^ →˙OH* + ˙OH^−^

35 ˙OH* + ˙O_2_ + ˙OH_˙_^−^ + ˙O_2_^−^ + e^−^ + h^+^ + Congo red dye → intermediate → CO_2_ + H_2_O

In the photocatalytic breakdown of Congo red dye, free radical electrons and holes can operate as photocatalytic agents. These holes formed by electron excitation from the valence band (VB) to the conduction band (CB) can behave as potent oxidizing agents. More reactive species are obtained because of a delay in the recombination of e^−^ and h^+^, which depends on the VB and CB. This can be mathematically expressed according to Mulliken's theory (eqn (36) and (37)).^65–67^

36 *E*_CB_ = *X* − *E*^c^ − 0.5*E*_bg_

37 *E*_VB_ = *E*_CB_ + *E*_bg_

In these equations, *E*_CB_ is conduction band energy, *E*_VB_ is valence band energy, *E*^c^ is free electron energy, whose magnitude is 4.5 eV, *X* is electronegativity of the photocatalyst and *E*_bg_ is band gap energy, respectively. The electronegativity of synthesized HAp is considered to be 5.89 eV, which is due to the geometrical mean of its constitutes, as illustrated in the literature.^65^ The calculated values of the conduction band (CB) and the valance band (VB) are listed in Table 4.

**Table 4:** Valance band (VB) and conduction band (CB) potentials

Sample name	Conduction band (eV)	Valance band (eV)
50% ethanol_HAp	−1.7075	4.4875
100% ethanol_HAp	−1.6655	4.4455
50% IPA_HAp	−1.695	4.475
Urea_HAp	−1.6675	4.4475
Naphthalene_HAp	−1.555	4.335

The synthesized HAps revealed higher negative potentials of the CB than O_2_/˙O_2_^−^ (−0.33 eV) and more positive potentials of the VB than OH/˙OH (1.99 eV), demonstrating that both radicals can be generated for photocatalysis of Congo red dye. The highest photocatalytic degradation or organic pollutant was found for the 100% ethanol-mediated HAp. This might be due to the growth of specific planes such as 110 and 202. The other crystallographic parameters were more or less similar for all types of synthesized HAps. Crystallite size calculated from a number of equations/models/plots carried good evidence for the formation of nano-crystallite HAp for all kinds of synthesized products. The estimated stress, strain, and energy density also showed significant variation for the various types of modifiers and solvent systems. Thus, different types of solvent systems and modifiers can be engaged for the wet chemical synthesis of HAp for the modification of crystallographic parameters and crystallite size. No significant variation was noticed in the surface morphology, optical bandgap, valence band energy, and conduction band energy for the variation of solvent systems and modifiers. The evaluation of photocatalytic performance of HAp compared to that of common metal oxide and sulfide photocatalysts is visualized in Table 5.

**Table 5:** The evaluation of photocatalytic performance of HAp compared to that of common metal oxide and sulfide photocatalysts^a^

Catalyst	Synthesis method	Conditions	Degradation (%)	Pollutant	References
100% ethanol_HAp	Wet chemical precipitation method	90 min, 10 ppm, 0.05 g	91.79	CR	This study
0.62Cu_HAp	Wet chemical precipitation technique	180 min, 20 ppm, 0.1 g	99	CR	2
TiO_2_	Hydrothermal method	210 min, 40 ppm, 1 g L^−1^	90	CR	68
CS/n-CdS	Simulating bio-mineralization process	180 min, 20 ppm, 1.5 g L^−1^	86	CR	69
BiOI/(BiO)_2_CO_3_	Chemical co-precipitation method	0.55 g L^−1^, 6 ppm, pH 6	81	SSZ	32
ZnO-based	Electrochemical method	240 min, 1 g L^−1^	83	CR	70
Co_3_O_4_/TiO_2_/GO	Sol–gel synthesis	90 min, 10 ppm, 0.25 g L^−1^	91	CR	71
Chitosan-zinc sulfide (CS-ZnS-NPs)	Co-precipitation method	100 min, 50 mg, pH 7, 10 ppm	96.7	Acid black 234	72
α-Fe_2_O_3_/Cu_2_O	Hydrothermal precipitation method	0.4 g L^−1^, 2 mg L^−1^, 45 min, pH: 6	90	MB	73

a CR = Congo red, MB = methylene blue, and SSZ = sulfasalazine.

## Conclusion

The results confirmed that the crystal structure of HAp can be modified by employing the wet chemical precipitation method. Additionally, the crystal size of the synthesized HAp was calculated using different models where all the calculated values lie in an acceptable range (1–150 nm), except for the straight-line method. SEM images showed spherical nanocrystals with distinctive uneven particles, likely due to particle aggregation. Furthermore, 100% ethanol_HAp shows the best result in terms of degradation percentage (91.79%) and degradation capacity (7.33%). Apart from this, the optical band gap of the synthesized HAp was measured at various values (5.89–6.19 eV) which may have little effect on the photocatalytic properties. From analyzing the estimated data, it's unequivocally stated that if the wet chemical precipitation method is followed using the above-mentioned chemical as well as synthesis parameters, the preferential growth of the (110 and 202) planes will be higher than that of any other plane for HAp nanocrystal synthesis. The specific planes may influence photocatalytic degradation of the organic pollutant. Thus from this research it is suggested to use organic modifiers and solvent for controlling crystallographic parameters to modify properties of samples.

## Author contributions

Md. Kawsar synthesized the hydroxyapatites and wrote the draft and original manuscript. Md. Sahadat Hossain conceived and designed the experiment and analysed the data. Sumaya Tabassum executed the photocatalytic experiment. Newaz Mohammed Bahadur and Samina Ahmed supervised the findings of this work. Samina Ahmed supervised the overall work, assisted in writing the final manuscript, and managed the required facilities.

## Data availability

Data will be made available on request.

## Conflicts of interest

There are no conflicts to declare.

## Supplementary Material
